# Bridging the Gap: Tackling Racial and Ethnic Disparities in Hypertension Management

**DOI:** 10.7759/cureus.70758

**Published:** 2024-10-03

**Authors:** Tabish W Siddiqui, Raqshan W Siddiqui, Syed Muhammad Hayyan Nishat, Asma A Alzaabi, Fatema M Alzaabi, Dana J Al Tarawneh, Abdallah Khan, Mohammed Abdul Muqsit Khan, Shiza W Siddiqui

**Affiliations:** 1 Internal Medicine, RAK Medical and Health Sciences University, Ras Al Khaimah, ARE; 2 Research, Dubai Medical College, Dubai, ARE

**Keywords:** black people, cardiovascular diseases, ethnicity, hypertension, racial groups, risk factors

## Abstract

Hypertension is a prevalent health concern with significant implications for cardiovascular disease risk, affecting diverse populations worldwide and imposing substantial health system burdens. This review article explores racial and ethnic disparities in hypertension prevalence, treatment, and management, highlighting the disproportionate impact on minority populations. Certain racial and ethnic groups in the United States exhibit higher prevalence rates of hypertension and related complications due to a confluence of genetic, social, and economic factors. Despite comparable treatment rates, blood pressure control is often less effective among these groups, partly due to less intensive treatment and systemic barriers to care. Different populations encounter unique challenges, with prevalence and control rates influenced by dietary habits, socioeconomic status, and healthcare disparities. This review summarizes current management practices and highlights the necessity for tailored approaches that consider ethnic-specific treatment responses. It underscores the importance of addressing socioeconomic and cultural barriers while incorporating both pharmacological and nonpharmacological treatments. Future research should focus on developing culturally relevant assessment tools, enhancing data collection, and evaluating interventions designed to mitigate these disparities. To promote health equity and optimize the management of hypertension in a variety of populations, it is imperative to address these inequities using individualized, evidence-based strategies.

## Introduction and background

The largest population-based risk factor for cardiovascular disease (CVD) is hypertension, which is defined as high blood pressure (BP) readings of ≥130/80 mmHg [1]. It is one of the most widespread chronic illnesses, with over one billion people globally suffering from hypertension, accounting for as much as 45% of the adult population [2]. This prevalence is consistent across all socioeconomic backgrounds [1]. Recent projections indicate that by 2025, the number of individuals suffering from hypertension could increase by 15% to 20%, increasing the total to around 1.5 billion [3]. The American Heart Association (AHA) reports that high BP is a leading preventable cause of CVD mortality and disease burden, accounting for 10.4 million deaths per year [4]. The current global economic burden of hypertension can be measured at 131 to 198 billion United States (US) dollars each year, based on an AHA estimate of 5768 US dollars per hypertension case [4].

Depending on whether a clear cause can be found, hypertension is divided into two categories: essential (primary) hypertension without a known cause and secondary hypertension with a known cause [5]. Although the exact underlying processes are yet unknown, essential hypertension affects over 90% of people with hypertension; it is believed to be the consequence of the combination of genetic and environmental factors [5]. Hypertension may result from variables that raise blood volume or reduce vasodilatation ability, which interact and coexist. These parameters are the focus of most antihypertensives [6].

Complications from hypertension-related morbidity and mortality are more common in racial minority populations [7]. Specifically, adults of African American descent are more likely than other racial minority groups in the US to have high BP [7]. In the US, the mortality rate from hypertension-related CVD is approximately four times higher for Black people than for White people [8]. These differences are not solely attributable to genetic predispositions but also reflect broader social determinants of health, including access to healthcare, lifestyle factors, and socio-economic status [9]. Significant gaps in addressing these disparities persist despite advances in medical treatments and public health measures [10]. Ethnic minorities may encounter obstacles in their quest for high-quality healthcare, have lower rates of early diagnosis, and receive interventions that are either ineffective or not culturally suitable [10].

Racial and ethnic minorities make up a substantial proportion of the US population and are more prone to suffer from hypertension and related complications. As such, addressing racial and ethnic disparities in the management of hypertension is imperative to improve public health outcomes. The objective of this review is to evaluate the efficacy of the existing management strategies while identifying the contributing causes of these inequalities. Furthermore, this research aims to provide useful insights and recommendations for better addressing the unique requirements of varied groups by combining the data from other studies. To advance health equity and raise the global standard of CVD management, it is imperative that knowledge in this field be enhanced.

## Review

Pathophysiology of hypertension

BP, diabetes mellitus, cigarette smoking, and high lipid levels are the primary modifiable risk factors for CVD [11]. Among these, high BP is associated with the strongest evidence of causation and is the most prevalent [11].

In hypertension, matrix metalloproteinases (MMPs) and tissue inhibitors of matrix metalloproteinases (TIMPs) have an impact on the remodeling of the vascular extracellular matrix (ECM) [12]. MMPs are a class of zinc-dependent endopeptidases that are necessary for many physiological functions, such as cell migration and tissue repair, but when they are dysregulated, they can cause fibrosis, tissue damage, and a weakening of the ECM [12]. In hypertension, this imbalance primarily affects the heart, kidneys, and blood vessels, leading to vascular damage [13]. Research has shown elevated levels of MMP-2, MMP-9, and TIMP-1 in hypertensive individuals, with MMP-9 playing a significant role in vascular remodeling and elevated BP [14]. An imbalance between MMPs and TIMPs, such as increased MMP-1 and subsequent collagen degradation, exacerbates arterial hypertension [14]. On the other hand, TIMP-3 has been linked to preventing angiotensin II-induced degradation of the arterial ECM. MMPs also have an impact on vascular smooth muscle cells (VSMCs) by stimulating growth factors that aid in the cells' proliferation [15]. MMP-2, in particular, can influence BP regulation by degrading vasodilator peptides, contributing to hypertension [14]. Elevated MMP-9 levels have also been associated with hypertensive complications like intracranial hemorrhage [16]. Thus, MMPs and TIMPs are crucial in regulating ECM metabolism and preserving vascular integrity in hypertension.

**Figure 1:**
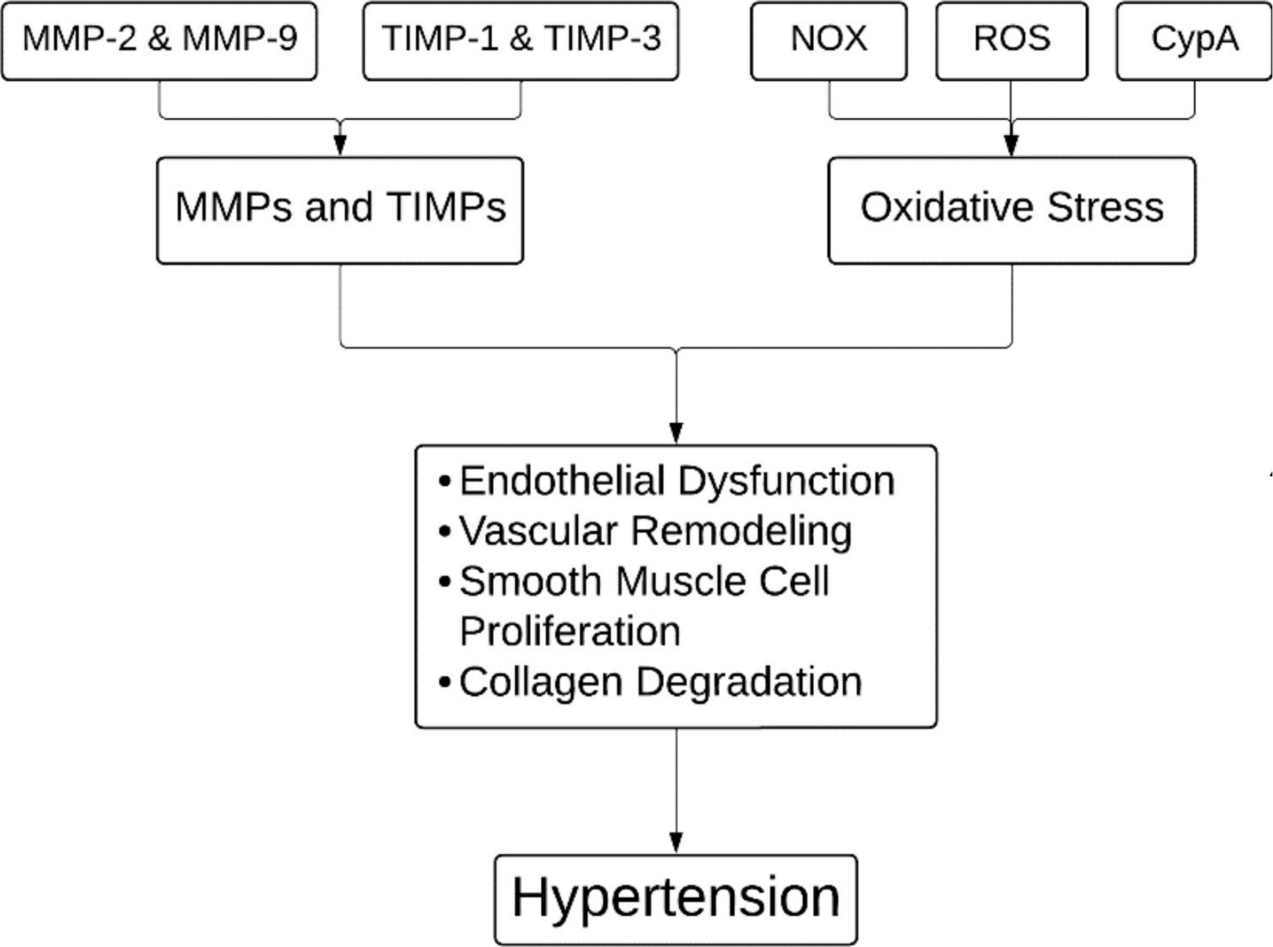
Pathophysiology of hypertension. MMPs: matrix metalloproteinases; TIMPs: tissue inhibitors of matrix metalloproteinases; NOX: NADPH oxidases; ROS: reactive oxygen species; CypA: cyclophilin A. Image credits: Tabish Siddiqui.

Increased BP, endothelial dysfunction, and vascular remodeling are associated with oxidative stress, which is defined by a decrease in antioxidant defenses and an increase in reactive oxygen species (ROS) [17]. ROS, mostly generated by nicotinamide adenine dinucleotide phosphate (NADPH) oxidases (NOX), contribute to vascular injury by inducing the proliferation of VSMCs, ECM protein deposition, inflammation, and endothelial dysfunction [18]. Upon being triggered by ROS, cyclophilin A (CypA) intensifies vascular damage by drawing in inflammatory cells and initiating MMPs [17]. Studies have demonstrated how NOX enzymes contribute to hypertension [19]. While NOX2 leads to endothelial dysfunction and vascular remodeling, overexpression of NOX1 in vascular smooth muscle enhances superoxide generation and exacerbates hypertensive responses [19]. On the other hand, under stressful circumstances, NOX4 plays a protective role by improving vasodilator function and BP management (Figure 1) [20].

Diagnosing hypertension

The American College of Cardiology (ACC) recommends obtaining at least two office measures at two distinct times to diagnose hypertension [1]. The European Society of Hypertension (ESH) recommends taking three office BP readings no more than one to two minutes apart. Additional measurements are only required if the first two readings deviate by more than or equal to 10 mm Hg [1]. The average of the previous two measures of BP is then noted [1]. The 2017 AHA and ACC recommendations define hypertension as BP greater than 130/80 mmHg [11]. Hypertension is defined as BP readings of ≥140/90 mm Hg according to the 2018 guidelines from the European Society of Cardiology (ESC) and the ESH, as well as the 2020 World Health Organization (WHO) and International Society of Hypertension (ISH) [1,21].

Variations in hypertension

Racial and ethnic disparities during the course of hypertension are well-known in the US. Black people have the highest prevalence of hypertension, with 59% of them suffering from high BP or using antihypertensive medication [22]. According to the National Health and Nutrition Examination Survey (NHANES) statistics, compared to White people, the rates of hypertension control are lower among African Americans, Hispanic Americans, and Asian Americans [23]. Nevertheless, compared to Black, White, and Hispanic individuals, Asian adults have been reported to have a higher likelihood of uncontrolled hypertension [8]. Compared to White adults, Black adults have a greater lifetime risk of hypertension [8]. Adults of Black, Hispanic, White, and Asian descent have a 40-year risk of developing hypertension of 93%, 92%, 86%, and 84%, respectively, according to the Multi-Ethnic Study of Atherosclerosis [24]. Black individuals in Europe experience higher rates of early and severe hypertension than White individuals, increasing the risk of developing end-organ damage [25]. When it comes to the development of end-stage renal disease and debilitating stroke, Black men are four times more likely to suffer from these conditions than White men [8]. Black people also have a greater prevalence of left ventricular hypertrophy and a four-to-five-fold increased risk of dying from hypertension-related complications when compared to white persons [8]. There is variation in the frequency and management of hypertension among Hispanic communities [26]. According to the Hispanic Community Health Study, Mexican Americans had lower six-year probabilities of having hypertension than Cuban and Dominican Americans [26]. The prevalence and management of hypertension vary among Asian American communities as well [27]. Approximately 64% of people with hypertension receive therapy, with varying incidence rates across Chinese, Korean, and Vietnamese subgroups [27]. These racial and ethnic groups continue to have greater rates of hypertension prevalence and poorer rates of control, despite differences in genetic and socioeconomic backgrounds [26,27].

Compared to people of other ethnicities (38.6% in Whites, 25.9% in Mexican Americans, 30.1% in other Hispanics, and 32.9% in other races), Black people had the highest prevalence of hypertension (46.1%), according to a recent study of the NHANES database by Fan et al. (2023) on the prevalence, treatment, and control rates of hypertension in the US [28]. Furthermore, it was observed that although the treatment rate for Black people was the greatest (64.1% compared to 61.0% for White people, 47.1% for Mexican Americans, 51.5% for other Hispanics, and 56.7% for other races), the control rate was the lowest, at 49.7% [28]. In contrast, the control rates for Whites, Mexican Americans, other Hispanics, and other races were 55.7%, 50.1%, 52.9%, and 53.1%, respectively [28]. Black individuals had the highest prevalence rate (45.3%), followed by Asians (31.8%), Hispanics (31.6%), and Whites (31.4%), according to a comparable study by Aggarwal et al. (2021) [29]. Additionally, treatment rates were found to be similar for White and Black individuals (67.3% and 67.2%, respectively); however, adult Hispanic and Asian individuals had lower treatment rates (60.5% and 58.8%, respectively) [29]. In contrast to Whites (49.1%) and Hispanics (40.0%), Black people had among the lowest control rates (39.2%), trailing only Asians (37.8%) while having greater treatment rates [29]. Comparing hypertensive individuals from the NHANES database from 1988 to 1994 and from 1999 to 2004, Cutler et al. (2008) conducted a similar analysis [30]. Black people exhibited the highest prevalence, according to the findings, which were consistent with those noted earlier by Fan et al. (2023) and Aggarwal et al. (2021) [28,29]. Comparing Black people to their White and Hispanic peers, it was also noted that Black people had the greatest treatment rates [30]. It was also noted that the lowest control rates were recorded among Hispanics (16.2% and 24.3%, respectively, in 1988-1994 and 1999-2004) (Table 1) [30].

**Table 1:** Prevalence, treatment, and control of hypertension among US adults. NHANES: National Health and Nutrition Examination Survey.

Reference	Study design	Population and setting	Ethnic group	Prevalence of hypertension	Treatment rate	Control rate
Fan et al. (2023) [28]	Cross-sectional population-based survey	53,496 participants of the NHANES 1999 to 2018	White	38.60%	61.00%	55.70%
			Black	46.10%	64.10%	49.70%
			Hispanic (Mexican American)	25.90%	47.10%	50.10%
			Hispanic (other)	30.10%	51.50%	52.90%
			Other races	32.90%	56.70%	53.10%
Aggarwal et al. (2021) [29]	Cross-sectional population-based survey	16,531 participants of the NHANES 2013 to 2018	White	31.40%	67.30%	49.10%
			Black	45.30%	67.20%	39.20%
			Hispanic	31.60%	60.50%	40.00%
			Asian	31.80%	58.80%	37.80%
Cutler et al. (2008) [30]	Cross-sectional population-based survey	14,430 participants of the NHANES 1999 to 2004	White	27.40%	62.10%	36.80%
			Black	40.10%	65.10%	33.40%
			Hispanic (Mexican American)	27.10%	47.40%	24.30%
Cutler et al. (2008) [30]	Cross-sectional population-based survey	16,351 participants of the NHANES 1988 to 1994	White	23.30%	54.20%	27.30%
			Black	35.80%	54.80%	24.00%
			Hispanic (Mexican American)	25.00%	38.60%	16.20%

Risk factors and determinants

Ethnic minorities are more susceptible to hypertension and CVD due to a plethora of variables besides established risk factors, such as consuming diets rich in sodium, smoking, drinking alcohol, being overweight, having a family history of hypertension, and suffering from diabetes [8,31]. The prevention, treatment, and management of hypertension are greatly influenced by geographic, local, and socioeconomic characteristics [8]. The WHO defines social determinants of health (SDH) as “the non-medical factors that influence health outcomes” [32]. Together with a larger group of factors and institutions influencing the conditions of everyday life, these are the circumstances under which individuals are born, develop, work, live, and age [32]. The NHANES reports a substantial correlation between uncontrolled hypertension and not having access to regular healthcare and a lack of insurance [23]. Geographical and neighborhood-specific factors also come into play [33]. Living in communities of low socioeconomic status or in some parts of the US, such as the southeastern states, is associated with a higher risk of hypertension and inadequate medical care [33]. The onset of hypertension is influenced by marital status, social class, and level of education [34]. A higher risk of hypertension has been linked, for instance, to lower educational attainment, widowhood, and living in impoverished areas [34].

Food insecurity raises the risk of hypertension and has an impact on dietary quality [35]. In addition, those on the Supplemental Nutrition Assistance Program have lower-quality diets than their counterparts with greater incomes, indicating the influence of structural variables such as food assistance programs [35].

Nutritional practices play a major role, as different ethnic groups have different levels of potassium and sodium homeostasis [36]. For instance, black individuals are more likely to have increased salt sensitivity and lower plasma renin levels, which makes them more prone to developing hypertension [36]. The Dietary Approaches to Stop Hypertension (DASH) diet is one dietary strategy that is beneficial in lowering BP [37]. Dietary therapies have demonstrated effectiveness in lowering BP in these populations, such as potassium supplementation [37]. China and South Korea, two nations with high sodium intake, have reduced their sodium intake through the implementation of policies that have led to a reduction in the prevalence of hypertension [36].

Acculturation has an effect on the management of hypertension in US communities of foreign-born individuals [38]. Americans of foreign birth often have lower rates of hypertension than Americans of American birth; nevertheless, prolonged residency in the US is linked to a higher risk of hypertension [38].

Psychosocial stressors and maladaptive coping strategies are two ways that racism and prejudice contribute to hypertension [8]. It has been established that discrimination throughout one's lifetime raises the risk of hypertension [39]. Deficits in access to nutritious food and medical treatment are made worse by structural racism, which is defined by institutional and systematic biases [40].

**Figure 2:**
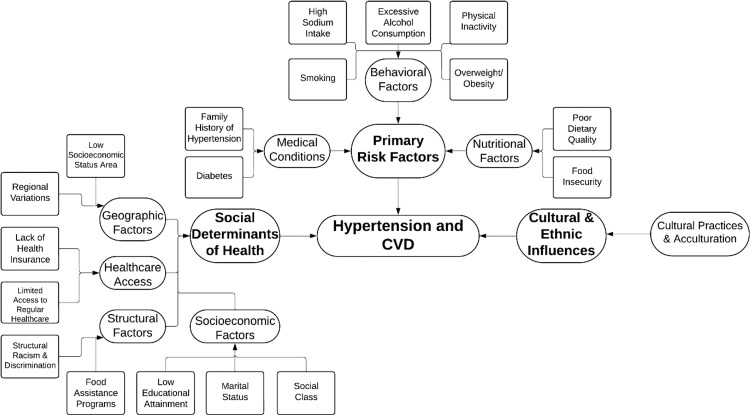
Risk factors and determinants contributing to hypertension and CVD. CVD: cardiovascular disease. Image credits: Tabish Siddiqui.

Figure 2 demonstrates the various risk factors and determinants involved in hypertension and CVD.

Management strategies

Racial and ethnic disparities in BP management persist despite similar rates of hypertension awareness and treatment [9]. NHANES data show that Black Americans have poorer rates of BP management than White Americans, in part because of less intensive treatment and greater rates of missed appointments [23]. Nonpharmacological therapies targeting lifestyle modifications are the cornerstone of initial care of hypertension in all cases [31]. Among these are quitting smoking, limiting alcohol intake, taking potassium supplements, losing weight, changing one's diet (such as switching to the DASH diet and consuming less sodium), and increasing physical exercise [31]. Even though these guidelines are widely applicable, racial and ethnic minority groups frequently encounter particular obstacles that make compliance difficult [8]. Disparities in resources, cultural norms, and socioeconomic status might all make it more difficult to carry out these lifestyle adjustments [33]. The use of various antihypertensive drugs, which may be tailored depending on ethnic-specific responses, is a key component of pharmacologic techniques for managing hypertension [36].

Nonpharmacological treatments, like following the DASH diet, losing weight, increasing physical activity, and reducing sodium intake, are essential for White populations [31]. Results from the Antihypertensive and Lipid-Lowering Treatment to Prevent Heart Attack Trial (ALLHAT) suggest that diuretics should be used as a pharmacological treatment for most people with high BP [41]. To achieve optimal BP management, combination treatment is often necessary, with one of the medications being a diuretic [41]. Additional medicines that are frequently used are angiotensin-converting enzyme (ACE) inhibitors, angiotensin receptor blockers (ARBs), calcium channel blockers (CCBs), and beta blockers [41].

Nonpharmacological treatments are similar for Black populations, but because of increased rates of salt sensitivity, there is also a focus on potassium supplementation [36]. When it comes to pharmacological treatment, thiazide diuretics are more beneficial for Black people than other antihypertensive classes; however, combined therapy is frequently required [41]. In addition to an emphasis on the use of antihypertensive medications from two or more different classes, for Black people without heart failure or renal impairment, the ACC/AHA guidelines suggest combining a thiazide-type diuretic with a CCB or an ACE inhibitor/ARB as first-line therapy [42]. Thiazide diuretics were found to be superior to other medication groups in the ALLHAT trial for preventing stroke in black patients [41]. Nevertheless, the ALLHAT trial did not demonstrate that other antihypertensives were superior to thiazide diuretics at lowering BP [41]. Loop diuretics are recommended for patients with heart failure or severe renal impairment (defined as an estimated glomerular filtration rate of less than 30 mL/min) [42]. For black patients with hypertension, combination therapy with an ARB and a CCB is advised as the first line of treatment, according to the International Society of Hypertension in Blacks (ISHIB) [43]. Since ACE inhibitors increase the risk of angioedema in black patients, ARBs are a better option for this group of people [36]. In the African American Study of Kidney Disease and Hypertension (AASK) trial, it was revealed that for Black individuals with non-diabetic renal disease, ACE inhibitors were more effective at slowing the course than other antihypertensives [44].

Given the prevalence of salt-sensitive hypertension in Japan and the Japanese Society of Hypertension's (JSH) current recommendation of diuretics as a first-line treatment, certain studies have suggested that Japanese patients might benefit from diuretic monotherapy [45]. It has been demonstrated that patients from South Asian backgrounds respond very well to ACE inhibitor/ARB treatment [8]. However, East Asians tend to favor ARBs over ACE inhibitors due to the latter's association with a higher prevalence of bradykinin-induced cough [36]. Despite the paucity of published research on the effectiveness of medications among Hispanic Americans, no clear hierarchy seems to exist in response to the different groups of antihypertensive drugs [46].

Single-pill combinations can improve adherence and help achieve BP control, whereas the polypill approach, which combines multiple antihypertensives into a single formulation, has shown promise in improving cardiovascular outcomes and managing BP, especially in low- and middle-income settings [42,47]. Successful control of hypertension requires a multidisciplinary, team-based approach, comprising physicians, pharmacists, nurses, dietitians, and community health workers collaborating in this strategy [48]. Better BP control and patient outcomes can result from integrating these professionals into an integrated care team that addresses hypertension management on both a pharmacologic and non-pharmacologic level [49]. Patients can track their BP at home, giving them vital information for therapy modifications [49]. If not adequately addressed, however, differences in broadband access and digital literacy could worsen already-existing health disparities [8].

Table 2 elucidates the various strategies used for BP control across different ethnic groups.

**Table 2:** Blood pressure control strategies across ethnic groups. DASH: Dietary Approaches to Stop Hypertension; ACE: angiotensin-converting enzyme; ARBs: angiotensin receptor blockers; CCBs: calcium channel blockers; BP: blood pressure; ALLHAT: Antihypertensive and Lipid-Lowering Treatment to Prevent Heart Attack Trial; ISHIB: International Society of Hypertension in Blacks; AASK: African American Study of Kidney Disease and Hypertension; JSH: Japanese Society of Hypertension.

Ethnic group	Nonpharmacological strategies	Pharmacological strategies	Effectiveness	Key studies
White	Diet modifications (DASH), weight loss increased physical activity, salt reduction	First-line agents: ACE inhibitors, ARBs, CCBs, and thiazide diuretics. Combination therapy: Often required for optimal control, involving combinations like ACE inhibitors + CCBs or ARBs + thiazide diuretics.	Effective BP control with standard treatments.	ALLHAT [41]
Black	Similar to Whites, with an additional focus on salt reduction and increased potassium intake	First-line agents: Thiazide diuretics are particularly effective. Preferred combinations: Thiazide diuretics + CCBs or ARBs, or ARBs + CCBs due to higher prevalence of salt sensitivity and increased risk of angioedema with ACE inhibitors.	Higher response to thiazide diuretics. Combination therapy is often required.	ALLHAT [41], ISHIB guidelines [43], AASK trial [44]
Hispanic	Diet modifications (DASH), weight loss increased physical activity	Pharmacological agents: ACE inhibitors, ARBs, and CCBs are commonly used. Combination therapy: Effective, with individualized approaches based on comorbidities.	Standard treatments are generally effective, but adherence may vary due to socioeconomic and cultural factors.	ALLHAT [41]
Asian	Similar to Whites and Hispanics, with an emphasis on reducing salt intake	Pharmacological agents: Diuretics are frequently recommended, especially in Japan. ACE inhibitors/ARBs: Effective, though ACE inhibitors are associated with a higher incidence of cough in East Asians.	Diuretics: Effective, particularly in managing salt-sensitive hypertension. ACE inhibitors: Effective but often replaced by ARBs due to higher cough rates.	JSH guidelines [45]

Future research directions

To further knowledge and develop better treatment approaches, future studies on hypertension in ethnic minorities should focus on several vital areas.

Tools for assessing CVD risk that are unique to ethnic minorities must first be created and validated [36]. Inaccurate risk prediction for Asian and Hispanic subgroups has been demonstrated by current techniques, such as the pooled cohort equations (PCE), which primarily represent the risk profiles of White and Black populations [50]. Better risk assessments and individualized preventative interventions will be possible with the creation and validation of new risk calculators catered to specific populations, based on comprehensive and representative datasets [51]. It is imperative that national health surveys like the NHANES have improved data-gathering capabilities [51]. The extensive data required for subgroup-specific analyses can be obtained by oversampling ethnic minorities, which will also advance our knowledge of the mechanisms by which racial and ethnic differences interact with socioeconomic and geographic factors to influence health outcomes [51]. The role that hereditary and environmental factors play in hypertension should also be extensively researched [36]. Research ought to look at genetic variants that impact the risk of hypertension in various ethnic groups as well as environmental factors such as food habits, body fat distribution, and socioeconomic stressors [36]. It is also critical to look at the social and regional factors that affect hypertension [8]. The effects of access to care, local health regulations, and rural versus urban environments on the course of hypertension should all be examined in research [8]. This includes researching how socioeconomic and regional inequities affect the availability and caliber of healthcare [52]. Additional research is necessary to determine the efficacy of healthcare interventions that are adapted to cultural and linguistic differences [52]. Assessing community-based initiatives, culturally aware educational resources, and language assistance can shed light on how to better treat hypertension across a range of ethnic groups [52]. Last but not least, long-term longitudinal research is required to monitor health outcomes and the efficiency of different hypertension management techniques over time [53]. By better addressing the requirements of ethnic minority communities, these studies will aid in the identification of enduring disparities and the improvement of management techniques [53].

Limitations

While offering insightful information, this review of hypertension among ethnic minorities also identifies a number of critical areas that require additional research and development. One significant drawback is the combination of different Asian populations with other ethnic categories, which may mask particular variations in the prevalence of hypertension and treatment outcomes among these groups. Although this method provides general insights, additional detailed information could improve our comprehension of problems unique to particular subgroups. There appears to be a deficiency of knowledge regarding the distinct hypertension profiles and difficulties faced by Asian and Hispanic populations. Research focused on these topics has the potential to greatly enhance management techniques.

There is also a need for standardized procedures in future research since variability in study methodologies and diagnostic criteria generates inconsistencies. Moreover, despite their crucial role in the outcomes of hypertension, socioeconomic and regional determinants are frequently underreported in the literature currently in publication. A more complete picture of the discrepancies and more informed interventions could result from taking these elements into account.

All things considered, these constraints offer prospects for improving research techniques and raising the efficacy of hypertension treatment in a variety of groups. It will be essential to address these issues to create healthcare plans that are more egalitarian and individualized.

## Conclusions

Significant differences in the prevalence and rates of control of hypertension between various racial and ethnic groups are indicative of a complex interaction between environmental, socioeconomic, and genetic factors. The findings emphasize the necessity for more precise subgroup analyses, particularly within diverse Asian and Hispanic populations, to better understand and address their unique hypertension challenges. Furthermore, enhancing the consistency of research methods and incorporating socioeconomic and regional factors will improve the validity and relevance of studies on hypertension.

Developing risk assessment instruments tailored to various ethnic groups and broadening data collecting to encompass a wider range of ethnicities should be the main goals of future research. This methodology will enable the development of culturally and regionally appropriate therapies, enhancing the management of hypertension and mitigating inequalities. It is imperative to address these research gaps and implement evidence-based, culturally sensitive strategies to advance health equity and optimize cardiovascular care across all communities.
